# Outcomes and associated factors of traumatic brain injury among adult patients treated in Amhara regional state comprehensive specialized hospitals

**DOI:** 10.1186/s12873-023-00859-x

**Published:** 2023-09-19

**Authors:** Solomon G/Michael, Bewuketu Terefe, Marye Getnet Asfaw, Bikis Liyew

**Affiliations:** 1 Department of Surgical Nursing, School of Nursing, College of Health Sciences, Aksum University, Aksum, Ethiopia; 2 Department of Community Health Nursing, School of Nursing, College of Medicine and Health Sciences, University of Gondar, Gondar, Ethiopia; 3 Department of Emergency and Critical Care Nursing, School of Nursing, College of Medicine and Health Sciences, University of Gondar, P.O.BOX 196, Gondar, Ethiopia

**Keywords:** Traumatic brain injury, Glasgow coma outcome scale, Amhara regional state

## Abstract

**Background:**

Globally, traumatic brain injury is the leading cause of death and disability which affects more than 69 million individuals a year.

**Objective:**

This study aimed to assess the outcome and associated factors of traumatic brain injury among adult patients treated in Amhara regional state comprehensive specialized hospitals.

**Method:**

Institutional-based cross-sectional study design was conducted from January 1, 2018, to December 30, 2020. A simple random sampling technique was used and a checklist was used to extract data between March 15 and April 15, 2021. The data were entered into Epi-data version 4.2 and exported to SPSS version 25 for analysis after being checked for consistency. Associated variables with outcomes of traumatic brain injury were determined by a binary logistic regression model. The degree of association was interpreted by using AOR and a 95% confidence interval with a *p*-value less than or equal to 0.05 at 95% CI was considered statistically significant.

**Result:**

In this study road traffic injury was the most frequent cause of traumatic brain injuries among adult patients, accounting for 181 (37.5%), followed by assault, accounting for 117 (24.2%) which affects adult age groups. One-third of the participant had a moderate Glasgow coma scale of 174(36%). Only 128(26.8%) patients arrived within one hour. One hundred sixty, 160 (33.1%) of patients had a mild traumatic brain injury, whereas, 149(36%) of patients had a severe traumatic brain injury. Regarding computerized tomography scans findings, the hematoma was the most common (*n* = 163, 33.7%). Ninety-one, 91(18.8%) of participants had cerebrospinal fluid otorrhea, and, 92(19%) were diagnosed with a positive battle sign. The overall prevalence of unfavorable outcomes after traumatic brain injury was found to be 35.2% (95%CI (30.8–39.1). Having additional Injury, hypoxia, time to hospital presentation after 24 h, severe Glasgow Coma Scale, moderate Glasgow Coma Scale, tachypnea, bradypnea, and cerebrospinal fluid Othorrhea, were factors associated with unfavorable outcomes.

**Conclusion and recommendation:**

In this study, the overall unfavorable outcome was experienced by about four out of every 10 victims of traumatic brain injury. Time of arrival > 24 h, low Glasgow coma scale, additional injury, Cerebrospinal fluid otorrhea, abnormal respiration, and hypoxia were significant predictors of unfavorable outcomes. To reduce the adverse effects of traumatic brain injury in adults, it is therefore desirable to guarantee safe road traffic flow and improve health care services.

## Introduction

Traumatic brain injury is a disruption of the brain structure with its function caused by the application of an external source which is manifest as confusion, loss of consciousness, coma, or seizure [1]. Based on the World Health Organization (WHO) report RTA is the 8^th^leading reason of death, and each year 1.35 million people are estimated to die due to RTA [2]. Globally, TBI is estimated that affects 69 million individuals each year; low and middle-income countries (LMICS) have three times higher burden of TBI than high-income countries, according to the WHO report. Internationally, TBI is the leading cause of death and disability [3–5].

In the United States of America, TBI is the commonest cause of mortality and disabilities. Annually, more than 2.8 million TBI cases were recorded with a 2% of death rate. Among those who survived, most of them experience several short and long-term impacts throughout their lives including thinking ability, physical activity, and loss of sensation like hearing and sight, emotional abnormality like depression. Furthermore, it has also impacted the lives of their families and thereby community [6, 7]. Besides, it results in immediate disruption of the brain function or it has lifelong mental or physical complications, and annually it is estimated worldwide greater than 50 million people may suffer from TBI complications and about half of the world population is expected to get one or more TBI within the lifetime [8]. Furthermore, studies showed that patients with a history of brain injury were significantly related to the chance of developing Parkinson's disease, epilepsy, stroke, and other neurological diseases this association is more common in different countries [9–11].

Additionally, death after the occurrence of TBI had been reduced; but the greatest number of severe TBI required long-term rehabilitation and these patients are suffering from the complication of TBI, which also has a consequence on social and economic costs [12].

Several studies suggested that early prevention of TBI is important to save lives, minimize disabilities and reduce healthcare-related costs. Regarding prevention strategies, giving priority to road traffic safety was found to be successful in some countries. Inappropriate use of motor vehicles in LMIC, infrastructural problems, and unable to use safety measures was found to increase the occurrence of TBI [13–15]. Likewise in our country, the pooled prevalence of TBI was reported at 20% and it is the leading reason behind mortality and disability, since RTA is the commonest cause of TBI the federal government has proclaimed the rules and regulations about the prevention strategies, but still mortality and severe disability were significantly associated with RTA [16, 17]. The global incidence rate of traumatic brain injury TBI is estimated at 200 per 100 000 people per year with wide variation in developed and developing countries [18]. During the COVID-19 pandemic era the challenges in treatment of the TBI in such countries were tremendous [19]. A similar recent report from Ethiopia also showed road traffic injury was the commonest cause of TBI. Increasing availability of radiological imaging scans and early resuscitations and surgical interventions are abetting in decreasing the mortality in recent years [20]. Prevention of hypoxia and maintaining cerebral perfusion pressure are imperative for favorable outcome. Kohler et al. have suggested narrative interviews, participatory diagramming and discrete event simulation as one possible suite of methods deliverable within an international partnership for boosting of outcome in developing countries like Ethiopia [21]. Different studies found in Africa and our country Ethiopia suggested that TBI is the leading cause of death and disability in addition to this the main reason for TBI was RTA [22–25]. As is the case globally, traumatic brain injury is a leading cause of morbidity and mortality in Ethiopia [8]. Road traffic incidents (RTI) are the greatest cause of TBI and substantial efforts have been undertaken to reduce RTIs in Ethiopia [26].

Despite these measures, the high incidence of TBI remains a major cause of disability that requires further study and public health intervention. As far as the researcher's search no related study has been undertaken so far in the study area. Therefore, this study aimed to assess the outcome and its associated factors of TBI among Patients Treated for traumatic brain Injury at the comprehensive specialized hospitals of the Amhara region, Ethiopia. 2021.

## Methods

### Study design and study period

An institutional-based retrospective cross-sectional study design was conducted on TBI patients who visited the comprehensive specialized hospitals of the Amhara region hospitals from January 1, 2018, to December 30, 2020,

#### Study setting

The study was conducted in the comprehensive specialized hospitals of the Amhara national regional state in Ethiopia. Amhara national regional state is one of Ethiopia's federal republics, with an approximate land area of 170,000 square kilometers [41]. The territory is divided into 12 administrative zones, three city administrations, and 83 districts. According to the Ethiopian twelve-month report for 2020, the region's total population projection is 22,191,890 people (11,317,864 men and 10,874,026 women), and the Amhara national regional health bureau's annual performance report shows the region has 81 hospitals, 858 health centers, and 3560 health posts. Among hospitals eight of them are comprehensive specialized hospital; these are the University of Gondar, Dessie, Felege-Hiwot, Tibebe-Ghion, Debre-Markos, Waldiy, Debre Tabor, and Debebirhan comprehensive specialized hospital which serves the population within the region [42]. These hospitals provide surgical, medical, pediatric, maternal, and other types of care to their patients. These hospitals have specialty units for cardiology, respiratory, neurology, dermatology, and sexually transmitted diseases, as well as gastroenterology, infectious diseases, orthopedics, gynecology and obstetrics, hematology, and intensive care units. Five comprehensive specialized hospitals were chosen by simple random lottery methods from a total of eight Comprehensive Specialized Hospitals those are University of Gondar, Felege-Hiwot, Tibebe-Ghion, Debre-Markos, and Debre-Berhan compressive specialized hospital. Thus, all those eight comprehensive specialized hospitals serve the population found in the region [27]. These hospitals provide specialized outpatient and inpatient services in different departments including emergency, surgical, internal medicine, gynecology & obstetrics, psychiatry, intensive care units (neonatal, pediatrics, and adult surgical and medical), ophthalmology, pediatrics, and oncology. Each hospital provides services for an estimated five million population. The hospitals have their organized trauma or surgical emergency departments, surgical wards, and surgical intensive care units [28]. ^The^ University of Gondar comprehensive specialized hospital is a referral tertiary care center with more than 600 beds located in the Amhara region, Northwest Ethiopia with a catchment population of more than 8 million people. In addition to general medical services, it provides advanced subspecialty surgical services including cardiothoracic surgery, neurosurgery, hepato-pancreatic-biliary surgery, pediatric surgery, urogynecology, and gynecologic oncology surgical services. There are around 20 general and subspecialist surgeons, 5 orthopedic surgeons, 20 general and subspecialist gynecologists/obstetricians, one anesthesiologist, and more than 30 anesthesia providers. It has also around 100 beds dedicated to surgical patients and 30 beds dedicated to gynecologic cases. It has 7 major operation theaters, 2 minor ORs, 2 obstetric ORs, and 2 urogynecology procedures. It has a 6 bedded surgical ICU dedicated to trauma and post-operative patients. On normal days, the hospital handles more than 50 OPD surgical cases, 5–6 elective operations, 4–5 elective gynecologic procedures, and 8–10 surgical and 5–10 obstetric emergency operations each day on average. The hospital uses an HMIS patient data recording system at surgery and gynecologic OPDs, at each ward, and all operation theaters record each patient profile, and the data is reported to the HMIS center monthly and annually.

### Population

#### Source population

The source population for this study was all patients presented with traumatic head injury attended in Amhara region comprehensive specialized hospitals.

#### Study population

Adult Patients presented in Amhara region comprehensive specialized hospitals, with the diagnosis of traumatic brain injury from January 1, 2018, to December 30, 2020, were the study population.

### Eligibility criteria

#### Inclusion criteria

All records of TBI patients who visited the comprehensive specialized hospitals of the Amhara region from January 1, 2018, to December 30, 2020, whose ages were above 18 years, were included in this study.

#### Exclusion criteria

Incomplete charts like missed outcome record, GCS score, time to arrival, conservative management, and loss from the record office due to consultation, transfer, or any other medical reason like neurological dysfunction before the occurrence of TBI, and death on arrival were excluded.

### Study variables

The dependent variable for this study is the Outcomes of Traumatic brain injury. The Independent variables are Socio-demographic factors( Age, sex, mode of arrival, source of referral, and area of residence), Patients condition at admission, Mechanism of injury, Time of arrival after injury, GCS on admission, the severity of the head injury, the occurrence of Co-existing injury, Type of co-existing injuries, comorbidity, pupillary reactivity, use of steroids, and Intervention taken, Clinical factors(Blood pressure, Respiratory rate, Pulse rate, O2 saturation, Blood glucose level, loss of consciousness, convulsion, battle sign, cerebrospinal fluid leakage, length of hospital stay and increase intracranial pressure), diagnostic factors, and CT-scan results.

### Operational definitions and definition of terms

*GOS*-Glasgow coma Outcome Scale was a multi-dimensional scale that assesses various aspects of outcome which are listed below:-

- *Death (GOS category 1)* – patients were certified for death
- *Vegetative state (GOS category 2)*–the patient exhibited no obvious cortical function
- *Severe disability (GOS category 3)* –patients were conscious but disabled, those patients could not perform any activity independently.
- *Moderate disability (GOS category 4)* – (disabled but independent) Those patients were independent as far as daily life was concerned the disabilities found include varying degrees of dysphasia and hemiparesis.
- *Good recovery (GOS category 5)*–Resumption of normal activities even though there may be minor neurological or psychological deficits
- *Unfavorable outcome* – was for the patient with GOS scored 1–3
- *Favorable outcome* – was for the patient with GOS scored 4 and 5
- *GCS*- was used to assess the neurological status of the patient.
- *Mild traumatic brain injury*:—was an injury to the head with a Glasgow coma scale between 13 and 15.
- *Moderate traumatic brain injury*:—was an injury to the head with Glasgow coma scales between 9 and 13.
- *Severe traumatic brain injury*:—was an injury to the head with a Glasgow coma scale less than or equal to 8.

### Sample size determination

The sample size was determined by using EPI info Version 7 statistical software to determine a single population proportion formula by considering the following assumptions. Based on the previous study, the proportion outcome of TBI 25.1% was taken from a cross-sectional study conducted in Nekemte in 2020 [29].

*P* = proportion of unfavorable outcome was (25%).

Z α/2 = the corresponding Z score of 95% CI,

d = Margin of error (4%) and.

*n* = required Sample size

$$\begin{array}{c}\mathbf{n}=\frac{{(\mathbf{Z}\mathrm{\alpha }/2)}^{2}\times \mathbf{p}(1-\mathbf{p})}{{(\mathbf{d})}^{2}}\\ \mathbf{n}=\frac{{(1.96)}^{2}\times (1-0.25)}{{(0.04)}^{2}}\\ \mathbf{n}=450\end{array}$$

The final sample size by adding a 10% nonresponse rate was 495.

For the second objective, by taking some of the significantly associated variables with an unfavorable outcome the sample size was determined through double population proportion formula using version 7 Epi info software (Table 1).

**Table 1:** sample size determination by using factors associated with TBI

Factors		Assumptions									
		Exposed	Non exposed	Power	Ratio	P1	CI %	P2	COR	N	Reference
age	< 20	61	9	80%	1:1	87.1%	95%				[24]
	Age > 60	14	39	80%	1:1		95%	26.4%	18.8	26	
Conservative treatment	No	104	20	80%	1:1	83.8%	95%				
	Yes	179	75	80%	1:1		95%	70.4%	2.179	336	
Time to arrival	< 24 Hour	226	16	80%	1:1	93.3%	95%				
	> 24 Hour	57	79	80%	1:1		95%	41.9%	19.5	32	

*Ratio* non- exposed to exposed, *P1*% of outcome in the exposed group, *P2*% of outcome in the unexposed group, *n* sample size

#### Sampling technique and procedures

**Fig. 1:**
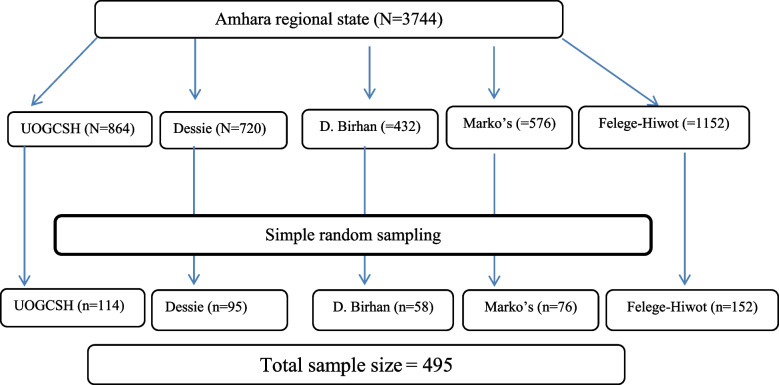
Sampling procedure for the outcome and its associate factors of TBI among adults who were treated for a head injury at comprehensive specialized hospitals Amhara region

The sample was selected with a simple random sampling technique thorough review of the patient's chart which is diagnosed with TBI for the last three years. The patient's medical record number was taken from the hospital ward registration book as a frame; the sample was proportionally allocated for each hospital. The final sample size of four hundred ninety-five was selected by using a simple random sampling method through the lottery method (Fig. 1).

### Data collection tools and procedures

The outcome and explanatory variables were collected through data extraction tool which was adopted from different literature [30–35]. And the data was recorded on structured checklists through a retrospective review of the patient charts. The data was collected by ten BSc nurses, after receiving one-day training on data collection tools and techniques. All the variables of interest were assessed accordingly. The questionnaire comprises five parts. Those socio-demographic factors, patient condition at admission, clinical profile, diagnostic factors, and outcome of TBI, Glasgow coma outcome scale (GOS) were used to assess the patient's outcome. This is a multidimensional outcome assessment scale it assessed the different aspects of outcomes. It has five categories based on that, GOS of I-III was considered as the unfavorable outcome, and GOS of IV, V was considered as a favorable outcome for statistical analysis which is taken through reviewing different studies [30, 31, 35]. This outcome assessment scale was introduced by Jennet and Bond in 1975. It has been widely used to describe the outcome of patients with a head injury. It has a high degree of validity (80%) and reliability (95%). The extraction tools for the assessment of associated factors are adapted from different related literature [29, 31, 33, 36].

### Data quality control

Different measures were taken to assure the quality of the data. The first measure was giving training for data collectors (ten BSc nurses) on how to extract data using the data abstraction format prepared for the study. To assess the clarity of the variable, a pre-test was done at 5% (25 medical charts) of the calculated sample size two weeks before the actual data collection at the University of Gondar comprehensive specialized hospital, those charts were excluded from the final study to check the validity and reliability of the collected data. After the pre-test, Ambiguous words and concepts were corrected accordingly. Throughout the data collection, data collectors were supervised by five MSc nurses. The collected data were checked by the supervisor daily for completeness and finally, the collected data were reviewed and checked for completeness before data entry; the incomplete data was discarded.

### Data processing and analysis

Data was checked manually, coded, and entered into Epi-Data Manager version 4.2, and it was exported to SPSS version 25 for analysis. Cross-tabulation was done to assess the distribution of favorable and unfavorable outcomes and the data was processed by carrying out simple descriptive statistics. The frequency with percentage distribution was used for categorical variables. Model goodness-of-fit was checked by the Hosmer–Lemeshow test. After checking the multi-collinearity each independent variable with a *p*-value < 0.25 in the bivariate analysis was included in multivariable logistic regression to control confounders. And finally, the variables which have an independent association with outcomes of TBI were identified based on AOR, with 95% CI and *p*-value less than 0.05 to measure the strength of association and identify statistical significance. Finally, the data was presented in the form of text, tables, and charts.

## Results

### Socio-demographic characteristics of the study participants

A total of 495 adult charts with the diagnosis of traumatic brain injury (TBI) were reviewed by using data extraction tools and included in the analysis with a response rate of 483(97.5%). Out of the total respondents, 296(61.2%) were male. The majority of the study participants 169(35%) were in the age group of 24–34 years. More than two-thirds 293(60.7%) were urban residents. More than two-thirds 293(60.7%) were urban residents. Out of the total 483 trauma patients, 320(66.2%) were transported to the hospital by ambulance followed by Bajaj 141(29.2%). Two hundred twenty-nine (47.4%) study participants were referred from the Health center followed by hospital 199(41.2%) (Table 2).

**Table 2:** Socio-demographic characteristics of favorable and unfavorable outcomes among adult patients in comprehensive specialized hospitals of Amhara regional state, Ethiopia 2021 (*n* = 483)

Variables	Category	Favorable outcome N (%)	Unfavorable outcome N (%)	Total N (%)
Age	18–24 years	82(17%)	39(8.1%)	121(25.1%)
	25–34 years	108(22.3%)	61(12.7%)	169(35%)
	35–44 years	54(11.2%)	29(6%)	83(17.2%)
	45–54 years	34(7%)	20(4.2%)	54(11.2%)
	> 55 years	35(7.2%)	21(4.3%)	56(11.5%)
Sex	Male	186(38.4%)	110(22.5%)	296(61.2%)
	Female	127(26.5%)	60(12.6%)	187(38.8%)
Residence	Urban	195(40.4%)	98(20.3%)	293(60.7%)
	Rural	118(24.4%)	72(14.9%)	190(39.3%)
Source of referral	Self	34(7%)	21(4.4%)	55(11.4%)
	Health center	147(30.4%)	82(17%)	229(47.4%)
	Hospital	132(27.3%)	67(13.9%)	199(41.2%)
Mode of Arrival	Ambulance	214(44.3%)	106(21.9%)	320(66.2%)
	Bajaj	87(18%)	54(11.2%)	141(29.2%)
	Police car	12(2.5%)	10(2.1%)	22(4.6%)

### Patient’s condition on admission and treatment-related factors

A road traffic accident was the leading cause of injury 181(37.5%) followed by assault 117(24.2%). One-third 86(34.4%) of trauma patients sustained maxillofacial injury and nearly one-fourth 58(23.2%) of the study participants had an abdominal injury. Twenty-four (9.6%) of trauma patients had poly-trauma. In one hundred twenty-eight cases 132(27.3%) arrived at the health care facilities they received care from the scene greater than 24 h followed (by 26.8%) within 1 h, 106(21.9%), within 1–4 h, and 117(24.2%) 4–24 h from the incident regardless of the mode of arrival (Table 3).

**Table 3:** Patient's condition on the admission and treatment-related factors of favorable and unfavorable outcomes among adult patients treated in comprehensive specialized hospitals of Amhara regional state, Ethiopia 2021 (*n* = 483)

Variables	Category	Favorable outcome *N* = (%)	Unfavorable outcome *N* = (%)	Total
Mechanism of injury	Road traffic accident	109(22.5%)	72(15%)	181(37.5%)
	Assault	83(17.2%)	34(7%)	117(24.2%)
	Fall down	56(11.6%)	33(6.8%)	89(18.4%)
	Others	65(13.5%)	31(6.4%)	96(19.9%)
Time to arrival	< 1 Hour	95(19.6%)	33(6.9%)	128(26.8%)
	1–4 Hours	61(12.6%)	45(9.3%)	106(21.9%)
	4–24 Hours	76(15.7%)	41(8.5%)	117(24.2%)
	> 24 Hours	81(16.8%)	51(10.5%)	132(27.3%)
GCS on admission	< 8	91(20.2%)	58(15.8%)	149(30.8%)
	9–12	98(18.8%)	76(12%)	174(36%)
	13–15	124(25.7%)	36(7.4%)	160(33.1%)
Additional injury	Yes	151(31.3%)	99(20.5%)	250(51.8%)
	No	162(33.5%)	71(14.7%)	233(48.2%)
Type of additional injury	Maxillofacial	49(19.6%)	37(14.8%)	86(34.4%)
	Chest injury	37(14.8%)	25(10%)	62(24.8%)
	Abdominal injury	36(14.4%)	22(8.8%)	58(23.2%)
	Pelvic injury	12(4.8%)	8(3.2%)	20(8%)
	Polytrauma	17(6.8%)	7(2.8%)	24(9.6%)
Blood pressure on admission	Normal	135(28%)	49(10.1%)	184(38.1%)
	Hypertension	76(15.8%)	50(10.3%)	126(26.1%)
	Hypotension	102(21%)	71(14.7%)	173(35.8%)
Intervention taken	Conservative management	169(35%)	77(15.9%)	246(50.9%)
	Surgery	80(16.6%)	56(11.6%)	136(28.2%)
	Both	64(13.2%)	37(7.7%)	101(20.9%)
Steroidal	No	265(55%)	148(30.5%)	413(85.5%)
	Yes	48(10%)	22(4.5%)	70(14.5%)
O_2_ saturation	Normal	215(44.5%)	77(16%)	292(60.5%)
	Hypoxia	98(20.2%)	93(19.3%)	191(39.5%)
Respiratory rate on admission	12–20	155(32%)	38(8%)	193(40%)
	> 12	82(17%)	65(13.4%)	147(30.4%)
	< 12	76(15.7%)	67(13.9%)	143(29.6%)
Pulse rate on admission	60–100	146(30.3%)	70(14.4%)	216(44.7%)
	> 100	78(16.1%)	47(9.8%)	125(25.9%)
	< 60	89(18.4%)	53(11%)	142(29.4%)

### Clinical characteristics

A significant number of the respondents 314(65%) had a loss of consciousness. Of the total respondents, 91(18.8%) respondents had CSF otorrhea and from the total sample charts, 92(19%) were diagnosed with a positive battle sign (Table 4).

**Table 4:** Clinical characteristics of favorable and unfavorable outcomes among adult patients treated in comprehensive specialized hospitals of Amhara regional state, Ethiopia 2021 (*n* = 483)

Variables	Category	Favorable outcome *N* = (%)	Unfavorable outcome *N* = (%)	Total
Pupillary reactivity	Normal	159(33%)	62(12.8%)	221(45.8%)
	Unilateral abnormality	67(13.9%)	45(9.3%)	112(23.2%)
	Bilateral abnormality	87(18%)	63(13%)	150(31%)
Comorbidity	Hypertension	35(7.2%)	24(5%)	59(12.2%)
	Diabetes mellitus	34(7%)	21(4.4%)	55(11.4%)
	Others	18(3.7%)	13(2.7%)	31(6.4%)
	No known disease	226(46.8%)	112(23.2%)	338(70%)
Loss of consciousness	No	113(23.4%)	56(11.6%)	169(35%)
	Yes	200(41.4%)	114(23.6%)	314(65%)
Convulsion	No	201(41.6%)	102(21.1%)	303(62.7%)
	Yes	112(23.2%)	68(14.1%)	180(37.3%)
Increase intracranial pressure	No	172(35.6%)	86(17.8%)	258(53.4%)
	Yes	141(29.2%)	84(17.4%)	225(46.6%)
Battle sign	Positive	51(10.5%)	41(8.5%)	92(19%)
	Negative	262(54.2%)	129(26.8%)	391(81%)
CSF leakage	No leakage	230(47.7%)	97(20%)	327(67.7%)
	Rhinorrhea	31(6.4%)	34(7.1%)	65(13.5%)
	Othorrhea	52(10.7%)	39(8.1%)	91(18.8%)
Blood glucose level	< 200	86(17.8%)	56(11.6%)	142(29.4%)
	> 200	56(11.6%)	27(5.6%)	83(17.2%)
	Not investigated	171(35.4%)	87(18%)	258(53.4%)
Length of hospital stay	1–3 days	85(17.6%)	51(10.6%)	136(28.2%)
	4–6 days	69(14.3%)	38(7.9%)	107(22.2%)
	7–10 days	50(10.3%)	28(5.8%)	78(16.1%)
	> 10 days	109(22.5%)	53(11%)	162(33.5%)
CT-scan result	No CT-scan	52(10.8%)	27(5.6%)	79(16.4%)
	Normal CT-scan	43(8.9%)	18(3.7%)	61(12.6%)
	Hematomas	95(19.7%)	68(14%)	163(33.7%)
	Contusion	43(8.9%)	17(3.5%)	60(12.4%)
	Subarachnoid hemorrhage	47(9.8%)	19(3.9%)	66(13.7%)
	Others	33(6.8%)	21(4.4%)	54(11.2%)

### Outcomes of traumatic brain injury

**Fig. 2:**
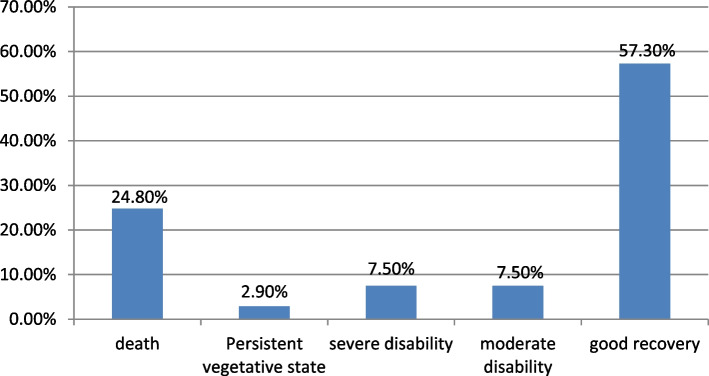
Outcome of Traumatic brain injury among patients treated in comprehensive specialized hospitals of Amhara regional state, Ethiopia 2021

From the total of 483 respondents, the outcome of the patient after traumatic brain injury on discharge was assessed based on GOS, and a large number of respondents, 277(57.3%) had a good recovery and among those unfavorable outcomes, 120(24.8%) died. For better analysis, the above outcomes are divided into two categories by using GOS such as favorable outcomes (64.8%) which consist of moderate disability and good recovery, and Unfavorable outcomes (35.2%) which consist of death, vegetative state, and severe disability (Fig. 2).

### Factors associated with outcomes of traumatic brain injury

As can be noted from the result of the bivariable analysis, eleven of the twenty-six variables show a significant association with the unfavorable outcome at the 95% level of significance. Those eleven variables were entered in the multivariable logistic regression at 0.25 level of significance multivariable logistic regression was done by taking eleven variables into account simultaneously. Five variables that showed significant association with unfavorable outcomes in the bivariable analysis could not persist as significant in the multivariable analysis. In the multivariable logistic regression analysis, after controlling the possible confounders; additional injury, low GCS on admission, abnormal respiration (tachypnea and bradypnea), and time of arrival after 24 h, hypoxia, and CSF otorrhea were statistically significant with the unfavorable outcome at *p*-value < 0.05.

Having additional injury showed a significant association with unfavorable outcomes. The odds of having an unfavorable outcome from TBI were 4.8 times higher in those who had additional injury than the counterpart [AOR = 4.844; 95% CI [2.532- 9.267]. This study showed that the odds of having unfavorable outcomes from TBI were 4.6 times more associated in those hypoxemic patients than those who were in normal oxygen status [AOR = 4.615; 95% CI (2.608–8.169]. Patients who were attending the hospital after 24 h after injury were two times higher to develop unfavorable outcomes than those who arrived less than one hour [AOR = 2.041; 95% CI [1.073- 3.882]. The odds of having an unfavorable outcome are two folds higher among patients with severe Glasgow coma scale [AOR = 2.142; 95% CI (1.196- 3.836], likewise the odds of having unfavorable outcome from TBI were 2.2 times higher for those patients with moderate Glasgow coma scale [AOR = 2.276;95% CI (1.308- 3.960] as compared to those who had mild Glasgow coma scale. The finding of this study showed that the odds of having unfavorable outcomes from TBI were 2.8 times higher in those who had CSF Othorrhea as compared to those without CSF leakage [2.884; 95%CI (1.417–5.870] (Table 5).

**Table 5:** Bivariable and multivariable logistic regression analysis result for significant variables among adult patients treated for traumatic brain injury attending comprehensive specialized hospitals of Amhara regional state, Ethiopia 2021 (*n* = 483)

Variables	Category	Favorable outcome	Unfavorable outcome	COR [95%CI]	AOR [95%CI]	*P*-value
Oxygen saturation	Normal	215(44.5%)	77(16%)	**1**	**1**	
	Hypoxia	98(20.2%)	93(19.3%)	2.65(1.8–3.89)	4.615(2.608–8.169)	.001
Sex	Male	186	110	1.25(0.85–1.84)	1.560(.985–2.469)	.058
	Female	127	60	**1**	1	
Mechanism of injury	RTA	109(22.5%)	72(15%)	1.39(0.82–2.33)	1.888(.891–4.002)	.097
	Assault	83(17.2%)	34(7%)	0.86(0.48–1.54)	1.892(.873–4.102)	.106
	Fall down	56(11.6%)	33(6.8%)	1.24(0.67–2.27)	1.313(.600–2.873)	.496
	Others	65(13.5%)	31(6.4%)	**1**	1	
GCS On admission	< 8	91(20.2%)	58(15.8%)	2.2(1.34–3.61)	2.142(1.196–3.836)	0.01
	9–12	98(18.8%)	76(12%)	2.67(1.66–4.3)	2.276(1.308–3.960)	.004
	13–15	124(25.7%)	36(7.4%)	**1**	1	
Pupillary reactivity	Normal	159(33%)	62(12.8%)	**1**	1	
	Unilateral abnormality	67(13.9%)	45(9.3%)	1.72(1.07–2.78)	.842(.430–1.647)	.616
	Bilateral abnormality	87(18%)	63(13%)	1.86(1.2–2.88)	.827(.423–1.618)	.579
Additional injury	Yes	151(31.3%)	99(20.5%)	1.5(1.03–2.18)	4.844(2.532–9.267)	.001
	No	162(33.5%)	71(14.7%)	**1**	1	
Battle sign	Positive	51(10.5%)	41(8.5%)	1.63(1.03–2.59)	1.472(.802–2.701)	.212
	Negative	262(54.2%)	129(26.8%)	**1**	1	
Respiratory condition	12–20	155(32%)	38(8%)	**1**	1	
	> 12	82(17%)	65(13.4%)	3.23(2.0–5.23)	6.879(3.160–14.9)	.001
	< 12	76(15.7%)	67(13.9%)	3.6(2.22–5.83)	7.266(3.367–15.6)	.001
Blood pressure status	Normal	135(28%)	49(10.1%)	**1**	1	
	Hypertension	76(15.8%)	50(10.3%)	1.81(1.12–2.94)	2.017(.890–4.573)	.093
	Hypotension	102(21%)	71(14.7%)	1.92(1.23–2.99)	.582(.299–1.133)	.111
CSF leakage	Normal	230(47.7%)	97(20%)	**1**	1	
	Rhinorrhea	31(6.4%)	34(7.1%)	2.6(1.51–4.47)	1.980(.975–4.020)	.059
	Othorrhea	52(10.7%)	39(8.1%)	1.78(1.1–2.87)	2.884(1.417–5.870)	.003
Time of arrival	< 1 h	95(19.6%)	33(6.9%)	**1**	1	
	1–4 h	61(12.6%)	45(9.3%)	2.12(1.22–3.69)	1.805(.943–3.454)	.074
	4–24 h	76(15.7%)	41(8.5%)	1.55(0.9–2.69)	1.015(.508–2.027)	.967
	> 24 h	81(16.8%)	51(10.5%)	1.81(1.07–3.08)	2.041(1.073–3.882)	0.03

## Discussion

In this study, the overall unfavorable outcome from TBI was 35.2%(n = 170) (95%CI(30.8–39.1), this study is consistent with the study conducted in Sudan at 31.4%. And with a study done at the University of Toledo Medical Center in Ohio 30% [37]. However, this finding was higher than a study conducted at Nekemte, Ethiopia 25.1% [29]. This discrepancy might be due to the difference in the number of hospitals included in the study and study period. The study conducted in Nekemte was conducted on a single referral hospital and it only includes two years of data, whereas the current study was done in all Amhara regional state comprehensive specialized hospitals and reviewed three years of data. This could cause a reasonable difference in the number of the outcome.

On the other hand, the finding of this study was lower than the study conducted in Kenya 55% [38], Austrian public hospitals 50% [39], Nepal 43.5% [40],, and Greece 40% [31]. This variation might be due to study subjects, the study subjects of the current study were all traumatic brain injury patients attending studied hospitals without specification of wards, but the study conducted in Kenya, Nepal, and Greece used study subjects under ICU department, patients under ICU have more chance of having an unfavorable outcome because, patients who are in life-threatening condition, serious infection, and severe injury are expected to admit to ICU department to get serious medical attention for 24 h and seven days and they would have a poor outcome after invasive procedures. In the current study, the likelihood of unfavorable outcomes from TBI is 4.6 times higher among patients with hypoxia than those who were in normal oxygen status. This finding agreed with the study done at Addis Ababa Tikur Anibesa specialized hospital, Ethiopia [41], Benin [42],, and Greece [31]. This is because hypoxia leads to a reduction in cerebral oxygen flow. So patients who had hypoxia may be complicated by cardiac arrest and small blood clots in the blood vessel consequently unfavorable outcomes [43].

And the odds of having unfavorable outcomes from TBI were 4.8 times higher in those who had additional injury than their counterpart. This study was consistent with the study employed in Nekemte referral hospital that suggested patients with additional injury had 5.8 times higher odds to have unfavorable outcomes [29]. And similar to a study conducted in Kenya that showed patients with additional injury had significantly associated with the unfavorable outcome. It might be the additional injury may cause patients to have other pathophysiologic reactions, such as neurologic shock due to pain from injuries, hypovolemia due to bleeding, hypotension, and other inflammatory reactions secondary to additional injury.

Regarding the mechanism of injury in TBI patients, road traffic injury was the commonest cause of traumatic brain injury which affected adult age groups 181(37.5%) followed by assault at 117(24.2%). These findings confirm what has been found and documented in South Africa, Zambia, Rwanda, and Europe [44–50].In contrast to this study, a study done in Norway the leading causes of TBI were falling down accidents, RTA, and assault [51].

In this study, 160 (33.1%) patients had mild traumatic brain injury the rest had moderate (*n* = 174, 30.8%)) to severe traumatic brain injury (*n* = 149, 36%). This study is comparable with a study conducted in Tanzania, England, and Nigeria [52–54]. Unlike that of Zambia where the number of operated patients was Moderate TBI [44].

In the current study, regarding CT scan findings, hematoma was the most common (*n* = 163, 33.7%). However, the study was conducted in Zambia, India, Tanzania, Ayder, Dilla University, and TASH Skull fracture followed by brain contusion [44, 52, 53, 55–57].

In this study, only 128(26.8%) patients came within one hour. Studies show that mortality will increase with incremental hours of patient arrival [58]. From the total of 483 participants, the outcome of the patient after traumatic brain injury on discharge was assessed based on GOS, and a large number of respondents, 277(57.3%) had a good recovery and among those unfavorable outcomes, 120(24.8%) died.

In this study, the overall unfavorable outcome from TBI was 35.2%( *n* = 170) (95%CI (30.8, 39.1). this study is lower than the Study conducted in Zambia, 58% of discharged TBI patients had a favorable GOS [44]. And higher than study conducted in Nekemte, Ethiopia (25.1%) were discharged with unfavorable outcome [59]. In this study, a relatively higher unfavorable outcome could be due to the absence of diagnostic imaging modality and Neurosurgery for moderate to severe TBI in the study area during the study period.

This study also revealed a severe and moderate type of head injury on admission was significantly associated with unfavorable outcomes. The odds of having unfavorable outcomes were 2.1 times higher for patients who had a severe Glasgow coma scale likewise, patients who had a moderate Glasgow coma scale had 2.2 times significantly associated with the unfavorable outcome as compared to those who had a mild Glasgow coma scale. This finding was in line with the study conducted in Addis Ababa [34], Nekemte referral hospital [29], Tunisia, Greece [31],, and the University of Toledo Medical Center in Ohio. For instance, the study done in Addis Ababa Tikur Anibesa and Nekemte referral hospitals showed that patients who had a low Glasgow coma scale on admission were 3.7 and 18.2 times higher chances to develop unfavorable outcomes respectively. This similarity might be due to an increased risk of secondary brain injury that leads to an increase in the risk of hematoma, brain edema, raised intracranial pressure, hypoxia, and hypotension for those patients who had severe and moderate Glasgow coma scale. Cerebrospinal fluid Othorrhea was significantly associated with unfavorable outcomes; the odds of having unfavorable outcomes from TBI were 2.8 times higher in those who had CSF otorrhea as compared to those without CSF leakage. This might be due to abnormal communication of the sterile subarachnoid space with the external environment, the patients may be at risk for meningitis, brain herniation, abnormal BP, and the first and most serious complication is spontaneous intracranial hypotension, where the pressure in the brain is severely decreased. Those complications may be the contributing factors to unfavorable outcomes [60].

This study finding showed that patients who were attending the hospital after 24 h after injury were two times higher chance to develop unfavorable outcomes than those who arrived less than one hour, this study is consistent with the study done at Dilla University referral hospital, Nekemte referral hospital [29], which indicates patients who arrived in hospital after 24 h had 4.7 and 16 times significantly associated with unfavorable outcome respectively as compared to those who arrived less than one hour and the study was similar with the study done in Kenya This can be explained timely diagnosis and management of the patients after traumatic brain injury is the key factor to prevent secondary brain injury through edema, hemorrhage, hypotension, and hypoxia.

Those patients who had tachypnea and bradypnea had a significant relationship with unfavorable outcomes. The odds of having unfavorable outcomes after TBI were 6.8 and 7.2 times high for those patients who had tachypnea and Bradypnea respectively. This might be because patients after traumatic brain injury have a chance to develop acute respiratory distress syndrome (ARDS), secondary to ARDS the patient may develop additional complications such as hypoxia, ischemia, and aspiration pneumonia. So having abnormal respiration is a leading cause to have unfavorable outcomes [61].

## Limitation

In the current study lack of some predictors such as the occupation of the patients, alcohol consumption before the injury, and nursing care for patients after TBI, which was a strong predictor of TBI, were not recorded. This may underestimate the role of those variables on the outcome of TBI.

## Conclusion and recommendation

In this study, the overall unfavorable outcome was experienced by about four out of every 10 victims of traumatic brain injury and time of arrival > 24 h, severe and moderate GCS on admission, having an additional injury, CSF otorrhea, abnormal respiration, and hypoxia were factors associated with the unfavorable outcome of traumatic brain injury among adults patients. Therefore our recommendation goes to upcoming researchers to use long-term longitudinal studies to better capture the recovery process and occurrence of late complications after TBI.

## Data Availability

The datasets generated and/or analyzed during the current study are not publicly available due (we didn’t have an open data sharing agreement between study participants and investigators for their data to be shared publicly). However, Data are available from the authors upon reasonable request.

## Abbreviations

TBI: Traumatic brain injury
GOS: Glasgow coma scale
AOR: Adjusted odds ratio
CI: Confidence interval
COR: Crude odds ratio
LMIC: Low- and middle-income countries
SPSS: Statistical Package for Social Sciences
RTA: Road traffic accident
WHO: World health organization
ICU: Intensive care unit
CSF: Cerebrospinal fluid
CSA: Central Statistical Agency
CSA: Central Statistical Agency
ARDS: Acute respiratory distress syndrome
TASH: Tikur anibesa specialized hospital
OR: Operation room
OPD: Outpatient departments
HMIS: Health Management Information Systems
BSc: Bachelor of Science
MSC: Master of Science
